# Diagnostic Accuracy of Multimodal Large Language Models for Four-Class Benchmark of Oral Autoimmune Blistering Diseases: A Multicenter Paired Study

**DOI:** 10.3390/diagnostics16172761

**Published:** 2026-08-28

**Authors:** Asmaa Abou-Bakr, Salma M Saad, Nevine H. Kheir El Din, Abdullah Bin Nabhan, Amal Bajonaid, Asma Saleh Almeslet, Fatma E. A. Hassanein

**Affiliations:** 1 Oral Medicine and Periodontology, Faculty of Dentistry, Galala University, Suez 43511, Egypt; 2 Oral Medicine and Periodontology Department, Faculty of Dentistry, Cairo University, Cairo 11553, Egypt; salma.saad@dentistry.cu.edu.eg; 3 Oral Medicine and Periodontology, Ain Shams University, Cairo 11566, Egypt; nevine.hassan@dent.asu.edu.eg; 4 Oral Medicine and Orofacial Pain, College of Dentistry, Prince Sattam Bin Abdulaziz University, Al-Kharj 11942, Saudi Arabia; a.binnabhan@psau.edu.sa; 5 Oral Medicine Department of Maxillofacial Surgery and Diagnostic Sciences, College of Dentistry, Jazan University, Jazan 45142, Saudi Arabia; abajonaid@jazanu.edu.sa; 6 Oral and Maxillofacial Surgery and Diagnostic Sciences, College of Medicine and Dentistry, Riyadh Elm University, Riyadh 12734, Saudi Arabia; asma.almeslet@riyadh.edu.sa; 7 Oral Medicine, Periodontology, and Oral Diagnosis, Faculty of Dentistry, King Salman International University, South Sinai 46612, Egypt

**Keywords:** autoimmune blistering diseases, oral diagnosis, large language models, artificial intelligence, diagnostic accuracy, clinical decision support

## Abstract

**Background/Objectives:** To compare the diagnostic performance of Claude Opus 4.7 and Gemini Pro 3 for the differential diagnosis of oral autoimmune blistering diseases (AIBDs) and evaluate their diagnostic reasoning, confidence, and calibration. **Materials and Methods:** This retrospective multicenter paired diagnostic accuracy study included 200 clinicopathologically confirmed AIBD cases (50 each of pemphigus vulgaris, mucous membrane pemphigoid, bullous pemphigoid, and linear IgA bullous dermatosis). Each case was independently assessed by both models using identical standardized clinical information and clinical photographic inputs. The task required forced-choice classification among the four predefined diseases. Histopathological and direct immunofluorescence findings were used exclusively to establish the clinicopathological reference diagnosis and were not provided to the AI models. The reference diagnosis was established by clinicopathological correlation. The primary outcome was diagnostic accuracy. Secondary outcomes included disease-specific diagnostic performance, Cohen’s κ, ROC analysis, calibration, confidence, reasoning quality, management recommendations, and error patterns. Pre-consensus inter-rater reliability of the two human assessors was also evaluated using Cohen’s κ for binary outcomes and weighted Cohen’s κ for the ordinal reasoning-quality score. **Results:** Claude achieved significantly higher diagnostic accuracy than Gemini (92.0% vs. 86.0%, *p* = 0.012), stronger agreement with the reference standard (κ = 0.893 vs. 0.813), and superior discrimination (macro-AUC 0.998 vs. 0.965). Claude demonstrated higher key diagnostic-feature identification (92.0% vs. 86.0%; *p* = 0.012) and higher clinical-reasoning scores (61.0% vs. 40.0% of responses rated good; Wilcoxon *p* < 0.001; *r* = 0.47), whereas management recommendations did not differ significantly (100.0% vs. 98.0%; *p* = 0.125). Calibration results were metric-dependent: Claude had a lower one-vs-rest Brier score (0.0468 vs. 0.0558), whereas Gemini had a lower expected calibration error (0.083 vs. 0.251). For both models, the predominant error was misclassification of linear IgA bullous dermatosis as mucous membrane pemphigoid. **Conclusions:** Both multimodal LLMs showed high performance in this controlled four-class benchmark, with Claude Opus 4.7 outperforming Gemini Pro 3 in overall accuracy and reasoning quality. However, these findings do not establish autonomous diagnostic capability, clinical effectiveness, or safety. The LABD–MMP misclassification and metric-dependent calibration highlight important limitations. The models should therefore be regarded as investigational adjunctive decision-support tools requiring clinician oversight and diagnostic verification. Prospective external and human-in-the-loop validation is required before clinical implementation.

## 1. Introduction

Accurate and timely diagnosis of autoimmune blistering disorders, most notably pemphigus vulgaris, mucous membrane pemphigoid, and bullous pemphigoid, continues to pose a formidable challenge in clinical practice. Characterized by significant phenotypic overlap, including mucosal erosions, cutaneous blistering, and pruritus, these rare immune-mediated diseases frequently present with overlapping clinical features, leading to delayed diagnosis, prolonged disease activity, and increased morbidity. Early diagnosis is important because timely management may prevent permanent tissue damage and improve patient outcomes [1,2,3].

The diagnostic gold standard incorporates clinical suspicion with histopathological examination and direct immunofluorescence; however, these investigations are invasive, require specialized expertise, and may not be readily available in all clinical settings, underlining the need for reliable ancillary diagnostic aids [2,4]. The four autoimmune blistering diseases evaluated—pemphigus vulgaris (PV), mucous membrane pemphigoid (MMP), bullous pemphigoid (BP), and linear IgA bullous dermatosis (LABD)—differ in their immunopathological mechanisms but may share overlapping oral and cutaneous manifestations. PV is an intraepithelial blistering disease primarily associated with autoantibodies to desmogleins, whereas MMP, BP, and LABD are subepithelial/subepidermal disorders involving distinct basement membrane zone targets. This clinical overlap, particularly between MMP and LABD, can make clinical differentiation challenging and underscores the importance of histopathological and direct immunofluorescence assessment [5,6,7].

Artificial Intelligence (AI) has emerged as a promising tool in oral medicine for diagnostic support [8,9]. Nonetheless, traditional AI approaches, mainly based on convolutional neural networks or image-based deep learning, primarily analyze isolated images and thus lack applicability to rare and diagnostically challenging diseases such as AIBDs. These methods are limited by relatively small datasets and their inability to incorporate heterogeneous clinical information, including medical history, clinical signs, histopathology, and immunopathology, which are essential for accurate diagnosis [10,11].

The recent release of multimodal large language models (MLLMs) is a major advancement, offering a new perspective on this integration challenge. Models such as Claude and Google’s Gemini are designed to jointly process and reason over multimodal clinical data within a single framework, thus emulating a clinician’s diagnostic workflow [12].

Recent studies have demonstrated their potential in interpreting medical images ranging from radiology to ophthalmology and producing coherent clinical reasoning [13]. However, evidence regarding their application in oral autoimmune blistering diseases remains scarce, and there is currently no comparison between state-of-the-art MLLMs for these conditions.

Although promising, the diagnostic performance of these multimodal LLMs may differ based on model architecture, multimodal reasoning capability, and calibration. Comparisons of these state-of-the-art models are needed to identify their strengths and limitations and determine their potential clinical utility in oral medicine. In addition to diagnostic accuracy, confidence calibration and clinical reasoning and management recommendations are becoming increasingly important for evaluating their clinical applicability.

In this regard, the present investigation aimed to fill this research gap by comparing the diagnostic performance of Claude Opus 4.7 and Gemini Pro 3 using clinically relevant standardized multimodal data obtained from clinicopathologically confirmed cases of oral autoimmune blistering diseases. This study differs from the existing literature by evaluating not only the diagnostic accuracy of these models but also their disease-specific performance, calibration, diagnostic confidence, clinical reasoning, and management decisions.

## 2. Materials and Methods

### 2.1. Study Design

This retrospective, multicenter, paired study evaluated the comparative performance of Gemini Pro 3 (Google DeepMind) and Claude Opus 4.7 (Anthropic) as investigational AI-based decision-support systems in a controlled, closed-set four-class benchmark of autoimmune blistering diseases (AIBDs) affecting the oral cavity. Specifically, the study evaluated model performance in classifying cases among four predefined AIBDs and generating clinical reasoning and initial management recommendations from standardized clinical case information. The study was not designed to evaluate autonomous medical diagnosis, prospective clinical effectiveness, patient benefit, or clinical safety. The study was conducted in accordance with STARD 2015 and the reporting guideline for diagnostic accuracy studies using artificial intelligence [14,15].

Archival cases were retrieved from the Faculty of Dentistry, King Salman International University (South Sinai, Egypt), the Faculty of Dentistry, Galala University (Suez, Egypt), and the Faculty of Dentistry, Cairo University (Giza, Egypt). Eligible cases were identified through a systematic review of electronic health records and institutional clinical archives and were consecutively included according to predefined eligibility criteria. All data were de-identified before analysis. Each case was independently evaluated by both AI models using identical standardized inputs and prompts, enabling direct paired comparison of diagnostic performance.

The study protocol was approved by the Institutional Review Board of King Salman International University (Approval No. IRB013-2025). The requirement for informed consent was waived because of the retrospective design and use of anonymized archival data. The study was conducted in accordance with the Declaration of Helsinki.

### 2.2. Study Population

Eligible cases were retrospectively identified from the electronic health records and clinical archives of the participating institutions. Consecutive patients with a definitive diagnosis of an autoimmune blistering disease (AIBD) involving the oral mucosa identified from institutional clinical archives and electronic health records between January 2025 and May 2025 were screened for eligibility. The reference diagnosis was established based on comprehensive clinicopathological evaluation, including clinical examination, histopathological findings, and direct immunofluorescence (DIF), with additional indirect immunofluorescence (IIF) and/or serological testing (ELISA) when available.

**Inclusion criteria.** Cases were included if they had (1) a confirmed diagnosis of pemphigus vulgaris (PV), mucous membrane pemphigoid (MMP), bullous pemphigoid (BP), or linear IgA bullous dermatosis (LABD); (2) complete clinical records; and (3) high-quality clinical photographs suitable for AI assessment.

**Exclusion criteria.** Cases with incomplete clinical documentation, inadequate image quality, uncertain diagnosis, duplicate records, or insufficient confirmatory investigations were excluded.

Representative case examples for each diagnostic category are provided in Supplementary Figure S1.

### 2.3. Sample Size

As this was a retrospective multicenter study, the final sample was determined by the number of consecutive eligible cases meeting the predefined inclusion criteria during the study period rather than by prospective sample-size recruitment. The final dataset comprised 200 biopsy-confirmed cases, including 50 cases each of pemphigus vulgaris (PV), mucous membrane pemphigoid (MMP), bullous pemphigoid (BP), and linear IgA bullous dermatosis (LABD). The balanced distribution across diagnostic categories was used to minimize class imbalance and facilitate comparable disease-specific evaluation of the two AI models.

For the primary paired comparison of overall diagnostic accuracy, the available sample was subsequently assessed in terms of precision and paired-test sensitivity. Precision was evaluated using the 95% confidence interval for the paired difference in diagnostic accuracy. A post-hoc sensitivity power analysis was additionally performed using an exact McNemar framework based on the observed discordant-pair proportions. This analysis was considered a sensitivity assessment rather than a priori power calculation.

### 2.4. Reference Standard

The reference diagnosis (ground truth) for each case was established through comprehensive clinicopathological evaluation by experienced oral medicine and oral pathology specialists at the participating institutions. The final diagnosis was based on the integration of clinical presentation, histopathological examination, and direct immunofluorescence (DIF) findings, in accordance with current international diagnostic criteria for autoimmune blistering diseases (AIBDs). Indirect immunofluorescence (IIF) and enzyme-linked immunosorbent assay (ELISA) results were incorporated when available to support the diagnosis. Only cases with a definitive diagnosis confirmed by clinicopathological correlation were included in the study. Histopathological and DIF findings were used exclusively to establish the reference diagnosis and were not provided as inputs to either AI model. The reference diagnosis served as the gold standard against which the diagnostic performance of Gemini Pro 3 and Claude Opus 4.7 was evaluated.

### 2.5. AI Models and Evaluation Procedure

Two state-of-the-art multimodal large language models (LLMs), Gemini Pro 3 (Google DeepMind) and Claude Opus 4.7 (Anthropic), were evaluated. Each model independently assessed all 200 cases under identical testing conditions using the same standardized prompt, case-level clinical information, and image inputs. For each case, the input included patient demographics, relevant medical history, clinical presentation, and a standardized high-resolution clinical photograph. Photographs were obtained using a high-resolution digital camera (Canon EOS 700D, Canon Inc., Tokyo, Japan) equipped with a Sigma 105 mm f/2.8 DG Macro lens and a Godox MF-R76 macro ring flash under consistent background and lighting conditions. Images were stored in JPEG format at full camera resolution and independently reviewed by two senior clinicians; photographs exhibiting blur, glare, or inadequate framing were excluded. The same clinical photograph and case-level clinical information were provided to both AI models for each case. Histopathological and direct immunofluorescence (DIF) images and results were not provided to the AI models and were not included in the case prompts; these findings were used exclusively to establish the clinicopathological reference diagnosis. All patient identifiers were removed before submission to the AI models.

The evaluated LLMs are proprietary, externally developed models. Their pretraining, fine-tuning, and internal validation datasets are not publicly accessible to the investigators; consequently, their composition, selection criteria, and performance characteristics could not be independently verified. The models were instructed to: (1) identify the most likely diagnosis from four predefined autoimmune blistering diseases (pemphigus vulgaris, mucous membrane pemphigoid, bullous pemphigoid, and linear IgA bullous dermatosis); (2) assign a probability (%) to each diagnostic category, with the four probabilities summing to 100%; (3) assign diagnostic confidence as a predefined ordinal category based on probability ranges: low (0–49%), moderate (50–79%), or high (80–100%); (4) describe the key clinical features supporting the diagnostic assessment; (5) recommend an appropriate initial management plan; and (6) clearly state relevant uncertainty or diagnostic limitations when appropriate.

Each case was evaluated independently by both models using separate chat sessions to prevent information carryover between cases. No follow-up prompts, iterative refinement, or response regeneration were permitted, and the first complete response generated by each model was retained for analysis. The complete standardized prompt is provided in Supplementary Part S1. All evaluations were performed through the respective provider web-based user interfaces rather than through API calls between February 2026 and June 2026. The model versions displayed at the time of evaluation were Gemini Pro 3 and Claude Opus 4.7. Because the web interfaces did not expose investigator-controllable temperature or top-*p* parameters or immutable backend build/snapshot identifiers, these parameters could not be independently fixed or recorded. Images were stored at full camera resolution before submission; any subsequent platform-side resizing or compression was controlled by the respective provider interfaces and could not be independently measured or modified by the investigators.

To maximize reproducibility, both models received the same predefined prompt, identical case-level clinical information, and the same clinical image for each case, with separate sessions used for each case and no follow-up prompts or regeneration. No missing-data imputation was performed because both models generated complete, interpretable outputs for all 200 included cases, with no indeterminate or non-responses. All model outputs were recorded in a standardized data-extraction form before statistical analysis.

### 2.6. Outcome Measures

**The primary outcome** was overall diagnostic accuracy, defined as the proportion of cases in which the model’s primary diagnosis agreed with the clinicopathological reference standard.

**Secondary diagnostic outcomes** included disease-specific sensitivity, specificity, F1-score, balanced accuracy, Cohen’s κ, and multiclass performance metrics (macro- and weighted precision, recall, and F1-score). Positive and negative predictive values were also calculated; however, because the study used an intentionally balanced dataset with equal numbers of cases in each disease category, these measures were interpreted as benchmark estimates rather than population-specific predictive values.

**Diagnostic discrimination** was evaluated using one-versus-rest receiver operating characteristic (ROC) analysis, with calculation of disease-specific and macro-averaged areas under the curve (AUC).

**Model calibration** was assessed using the mean one-versus-rest Brier score, top-prediction Brier score, expected calibration error (ECE), and calibration intercept and slope. Diagnostic confidence was analyzed separately as a three-level ordinal outcome (low, moderate, and high), corresponding to the predefined probability ranges of 0–49%, 50–79%, and 80–100%, respectively. Confidence was treated as a separately reported model output and was not derived post hoc from the maximum predicted probability among the four diagnostic categories. Confidence distributions between models were compared using the paired Wilcoxon signed-rank test, and diagnostic accuracy was summarized within each confidence category.

Human assessment of AI-generated responses was performed independently by two board-certified oral medicine specialists using predefined assessment criteria. Each response was assessed for key diagnostic-feature identification, appropriateness of the recommended initial management, and clinical reasoning quality. Key diagnostic-feature identification and management appropriateness were recorded as binary outcomes (correct/incorrect), whereas clinical reasoning quality was rated on a three-level ordinal scale (1 = poor, 2 = acceptable, 3 = good). Before consensus adjudication, inter-rater reliability was assessed using Cohen’s κ for the two binary outcomes and weighted Cohen’s κ for the ordinal reasoning-quality score, with corresponding 95% confidence intervals. Discrepant assessments were resolved by consensus, and the resulting consensus ratings were used for the comparative analyses of the AI models. No imputation was performed for missing human-assessment data.

### 2.7. Statistical Analysis

Statistical analyses were performed using R version 4.5.3 (R Foundation for Statistical Computing, Vienna, Austria), accounting for the paired evaluation of each case. The primary endpoint was overall diagnostic accuracy, defined as agreement between the top-ranked diagnosis and the clinicopathological reference diagnosis. The primary Claude–Gemini comparison used exact McNemar’s test, with paired odds ratio, absolute risk difference, and 95% confidence interval (CI). Disease-specific sensitivity, specificity, positive and negative predictive values, F1-score, and balanced accuracy were reported with Wilson 95% CIs; paired disease-specific comparisons used exact McNemar’s tests with Holm adjustment. Cohen’s κ with 95% CIs assessed agreement with the reference standard and between models. One-versus-rest ROC analysis used the probability assigned to each disease as the test variable, with that disease as positive and the remaining three as negative. AUCs and 95% CIs were estimated using DeLong’s method with logit transformation; paired DeLong comparisons used the same case-level predictions and Holm adjustment.

Calibration was assessed using one-versus-rest and multiclass Brier scores, top-prediction Brier score, ECE, maximum calibration error, and calibration plots with bin sizes. Calibration intercepts and slopes were reported only when estimable and were not interpreted as conventional calibration parameters when affected by complete or quasi-complete separation. Confidence thresholds were prespecified before model evaluation (low, 0–49%; moderate, 50–79%; high, 80–100%) for the separately generated model confidence output; these thresholds were used to standardize interpretation and were not selected based on observed model performance. Continuous confidence values were not retained as a separate analyzable outcome. Confidence and reasoning quality were analyzed as paired ordinal outcomes using the Wilcoxon signed-rank test, with effect size r for reasoning; key diagnostic-feature identification and management appropriateness were compared using exact McNemar’s tests. These were considered secondary/exploratory outcomes.

A binomial GEE with logit link, exchangeable working correlation, and case-level clustering assessed the model effect after adjustment for disease category. BP was excluded from the primary GEE because of complete separation, and a model-by-disease interaction was examined. This was a secondary analysis intended to estimate the model effect after disease adjustment and assess effect modification, rather than replace the prespecified paired comparison. Firth penalized logistic regression including all four diseases was performed as a separation-robust sensitivity analysis without within-case clustering.

A post-hoc precision assessment evaluated the 95% CI for the paired accuracy difference, and paired-test sensitivity was assessed using an exact McNemar framework based on the observed discordant pairs. Because the cohort was balanced across the four diagnoses by design, a prevalence-weighted sensitivity analysis was performed. Per-class accuracies and paired within-class differences were directly standardized to illustrative external disease frequencies under plausible clinical weightings, with variances obtained from within-class discordant pairs. The balanced-design comparison was retained as the primary analysis, and the standardized estimates were treated as a sensitivity analysis of generalizability. All tests were two-sided, with α = 0.05 for the primary endpoint; Holm-adjusted *p* values were reported for prespecified secondary inferential families.

## 3. Results

### 3.1. Study Cohort and Data Completeness

**Figure 1:**
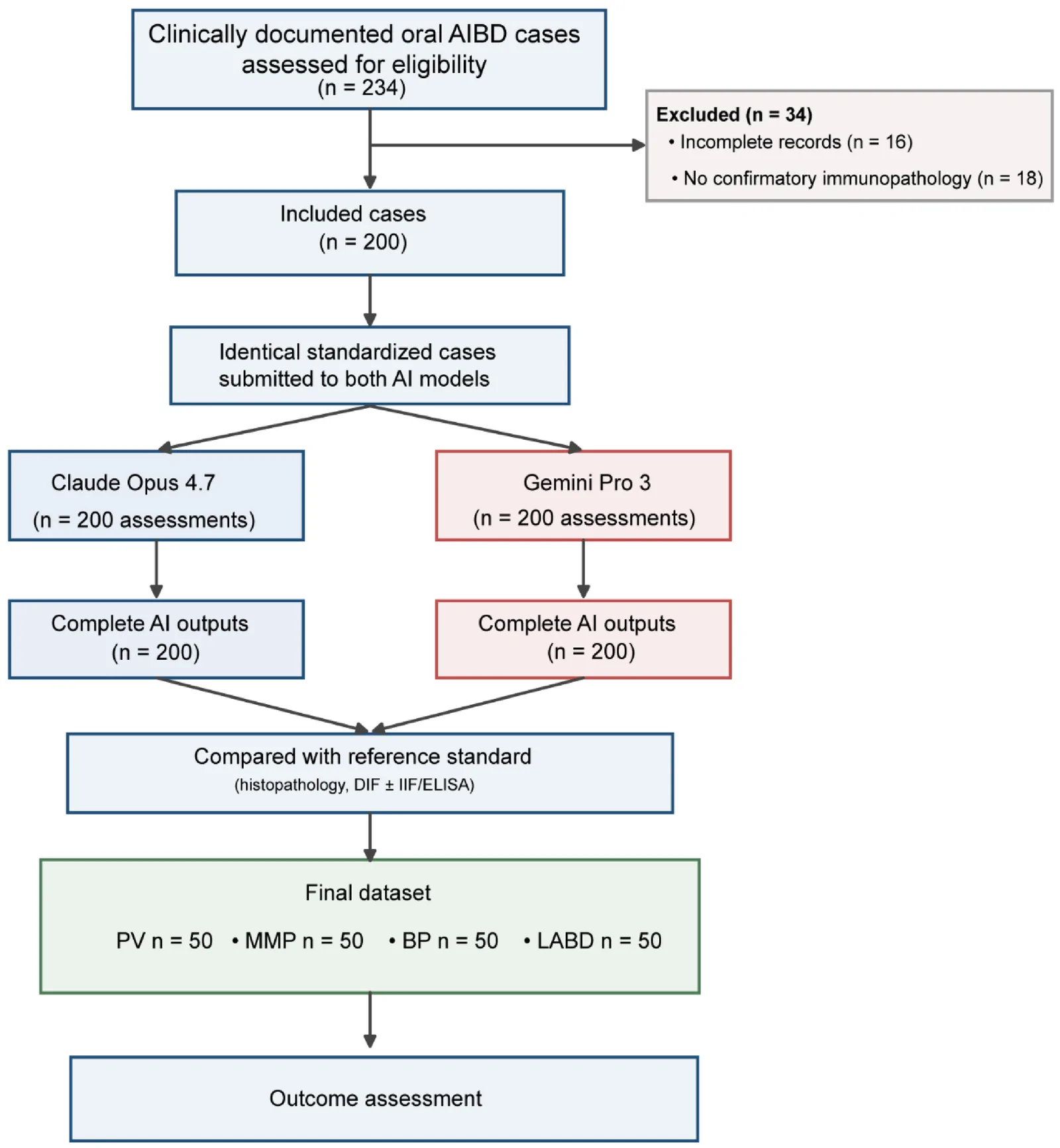
STARD flow diagram of case selection and analysis. Both index tests were applied independently to all 200 cases, with no indeterminate results and no missing reference-standard data.

A total of 234 clinically documented oral autoimmune blistering disease (AIBD) cases were screened for eligibility, of which 200 met the predefined inclusion criteria and were included in the final analysis. Thirty four cases were excluded because of incomplete clinicopathological documentation (*n* = 16) or lack of confirmatory immunopathological testing (*n* = 18). The final study cohort comprised 50 cases each of pemphigus vulgaris (PV), mucous membrane pemphigoid (MMP), bullous pemphigoid (BP), and linear IgA bullous dermatosis (LABD). Both AI models independently evaluated all included cases, generating complete diagnostic outputs without missing or indeterminate responses (Table 1; Figure 1).

### 3.2. Overall Diagnostic Performance

Claude Opus 4.7 correctly classified 184 of 200 cases (92.0%, 95% CI: 87.4–95.0), compared with 172 of 200 cases for Gemini Pro 3 (86.0%, 95% CI: 80.5–90.1), yielding an absolute paired accuracy difference of 6.0 percentage points (95% CI: 1.7–10.3). McNemar’s exact test indicated a significant difference between models (*p* = 0.012), with 16 discordant cases favoring Claude and 4 favoring Gemini (paired OR: 4.00; exact 95% CI: 1.29–16.44). Agreement with the reference standard was almost perfect for Claude (κ = 0.893, 95% CI: 0.843–0.943) and substantial for Gemini (κ = 0.813, 95% CI: 0.750–0.877). Inter-model agreement was 90.0% (κ = 0.866, 95% CI: 0.810–0.921). The estimated number needed to diagnose (NND) was 16.7, corresponding to approximately 17 cases evaluated with Claude rather than Gemini to obtain one additional correct classification (Table 2).

Because the cohort was intentionally balanced at 25% prevalence for each disease, a prevalence-weighted sensitivity analysis was performed to assess the effect of the artificial case distribution on overall accuracy. Under the primary balanced study design, the between-model difference was 6.0 percentage points (92.0% vs. 86.0%; 95% CI, 1.7–10.3). Under an illustrative clinical distribution of BP 45%, LABD 5%, PV 25%, and MMP 25%, accuracies were 96.8% and 94.0%, respectively (difference, 2.8 percentage points; 95% CI, −0.4 to 6.0). Under a second illustrative clinical distribution of BP 35%, LABD 5%, PV 30%, and MMP 30%, accuracies were 96.4% and 93.2%, respectively (difference, 3.2 percentage points; 95% CI, −0.6 to 7.0). These alternative distributions were illustrative rather than registry-derived and were used as sensitivity analyses of generalizability rather than estimates of population-level clinical accuracy (Supplementary Table S1).

### 3.3. Precision and Post-Hoc Power Assessment

The 95% confidence interval for the primary paired difference had a half-width of approximately 4.5 percentage points, indicating reasonable precision for estimation. A post-hoc sensitivity power analysis based on the observed discordance structure estimated approximately 73% power at *n* = 200. Under the same discordance assumptions, approximately 250 and 300 paired cases would provide 80% and 90% power, respectively.

### 3.4. Disease-Specific Diagnostic Performance

**Figure 2:**
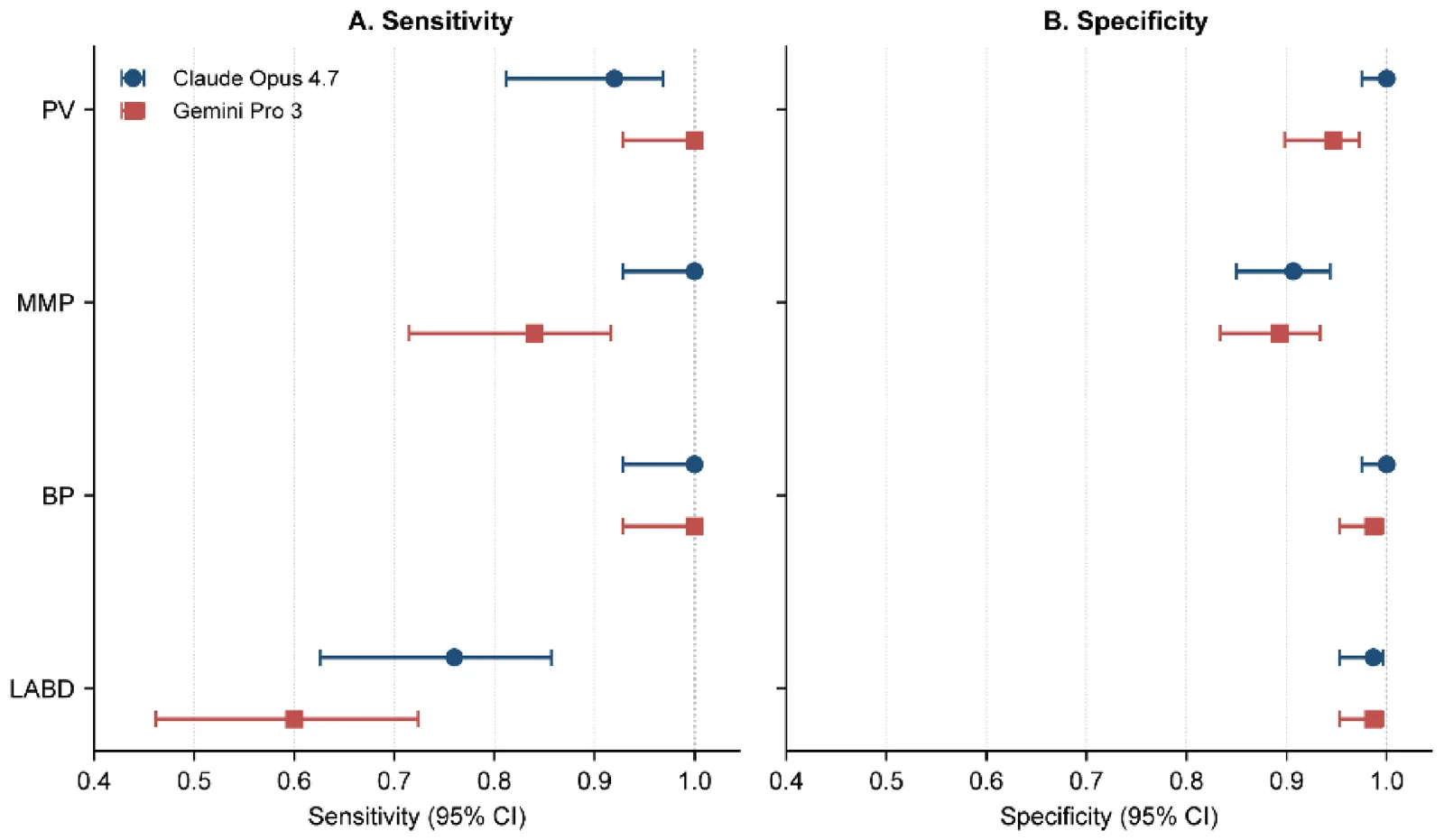
Disease-specific sensitivity (**A**) and specificity (**B**) of Claude Opus 4.7 and Gemini Pro 3 for the four autoimmune blistering diseases. Points indicate point estimates; error bars represent 95% Wilson confidence intervals.

Disease-specific diagnostic performance varied across the four autoimmune blistering disease subtypes. Bullous pemphigoid (BP) was identified with perfect sensitivity by both models, with Claude additionally achieving perfect specificity. Gemini generated two false-positive BP classifications among non-BP cases, resulting in a specificity of 98.7% and an F1 score of 0.980, whereas Claude achieved 100.0% specificity and an F1 score of 1.000. The greatest between-model differences were observed for linear IgA bullous dermatosis (LABD) and mucous membrane pemphigoid (MMP). Claude Opus 4.7 showed higher sensitivity for LABD (76.0% vs. 60.0%) and achieved perfect sensitivity for MMP (100.0% vs. 84.0%), although this was accompanied by a modest reduction in specificity (90.7%) due to preferential misclassification of some LABD cases as MMP. Disease-specific McNemar analyses demonstrated significant advantages for Claude in the diagnosis of MMP (*p* = 0.008) (unadjusted *p* = 0.008; Holm-adjusted *p* = 0.031) and LABD (unadjusted *p* = 0.008; Holm-adjusted *p* = 0.031), whereas no significant differences were observed for PV (unadjusted *p* = 0.125; Holm-adjusted *p* = 0.250) or BP (*p* = 1.000, with no discordant pairs). Bootstrap confidence intervals closely paralleled the Wilson confidence intervals across all performance metrics, indicating robust and stable estimates (Table 3). Bootstrap confidence intervals were broadly consistent with the corresponding Wilson intervals across the reported performance measures (Table 3). The corresponding forest plot illustrates the between-model differences in sensitivity and specificity across the four disease categories (Figure 2).

### 3.5. Multiclass Classification Performance

Beyond disease-specific diagnostic performance, multiclass classification metrics were evaluated to assess the overall ability of each model to correctly distinguish among the four autoimmune blistering diseases. Claude Opus 4.7 achieved higher multiclass performance metrics than Gemini Pro 3 across the measures reported in Table 4. Claude achieved higher macro-averaged F1-score (0.920 vs. 0.854), macro-averaged precision (0.933 vs. 0.871), macro-averaged recall (0.920 vs. 0.860), and balanced accuracy (92.0% vs. 86.0%). Weighted F1-scores were identical to the corresponding macro-averaged F1-scores (0.920 vs. 0.854), reflecting the equal number of cases in each diagnostic category.

**Figure 3:**
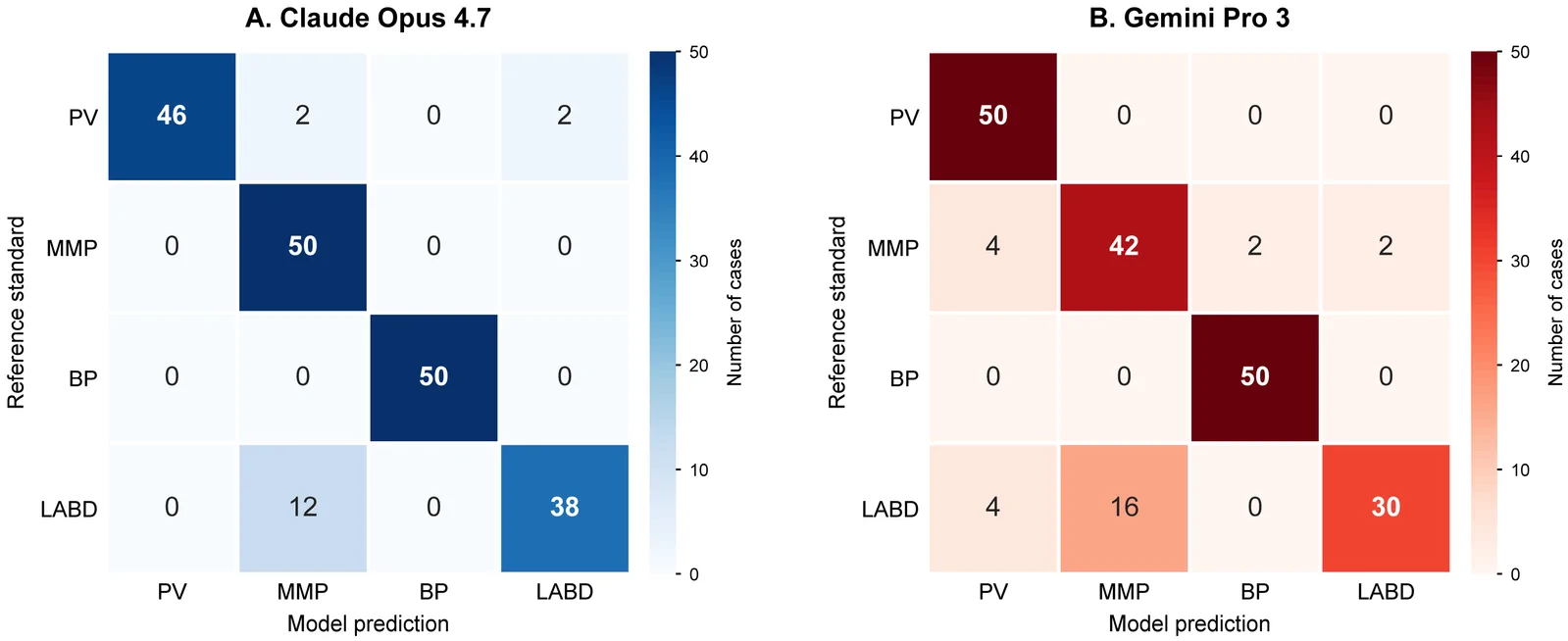
Confusion matrices for Claude Opus 4.7 (**A**) and Gemini Pro 3 (**B**) in the four-class diagnostic benchmark. Rows represent the reference-standard diagnosis and columns represent the predicted diagnosis. Color intensity indicates the number of cases in each cell, with darker shades representing higher case counts.

Confusion matrix analysis further illustrated multiclass classification performance of Claude Opus 4.7 (Figure 3). Both models correctly classified all bullous pemphigoid (BP) cases, whereas most classification errors occurred in linear IgA bullous dermatosis (LABD). The predominant error pattern for both models was misclassification of LABD as mucous membrane pemphigoid (MMP), although this occurred less frequently with Claude than with Gemini. In this balanced four-class benchmark, overall accuracy and balanced accuracy were identical for each model (92.0% for Claude and 86.0% for Gemini).

### 3.6. Diagnostic Discrimination

Receiver operating characteristic (ROC) analysis demonstrated excellent diagnostic discrimination for both multimodal LLMs (Table 5). Claude Opus 4.7 achieved a higher macro-averaged area under the ROC curve (AUC) than Gemini Pro 3 (0.998 vs. 0.965). Disease-specific one-versus-rest ROC analyses showed that Claude achieved perfect discrimination for pemphigus vulgaris (PV), mucous membrane pemphigoid (MMP), and bullous pemphigoid (BP) (AUC = 1.000 for each), while maintaining near-perfect discrimination for linear IgA bullous dermatosis (LABD) (AUC = 0.993; 95% CI, 0.978–0.997). The corresponding AUCs for Gemini Pro 3 were 0.992 (95% CI, 0.980–0.997), 0.930 (95% CI, 0.854–0.968), 0.987 (95% CI, 0.948–0.997), and 0.952 (95% CI, 0.896–0.979), respectively.

**Figure 4:**
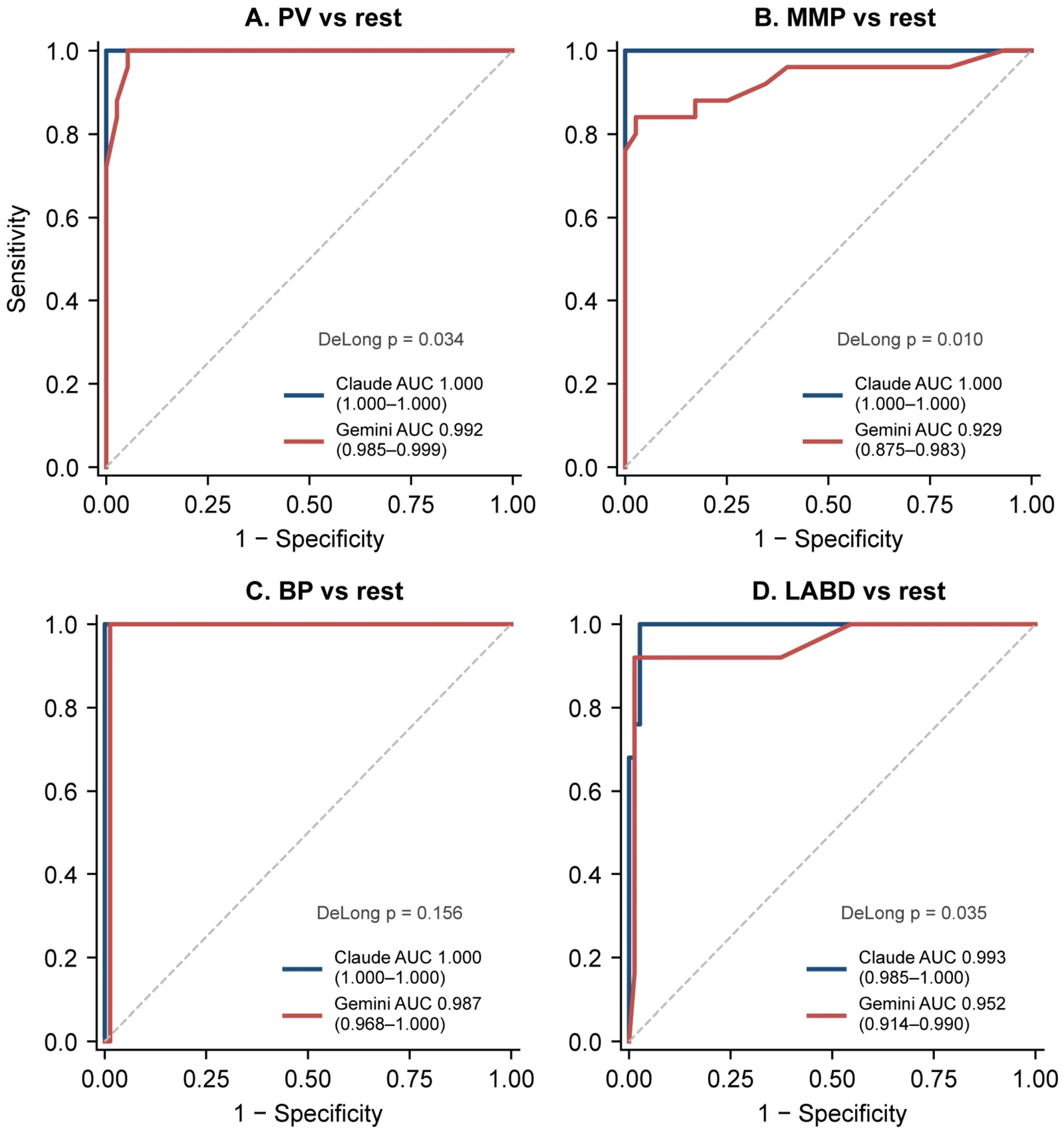
One-versus-rest receiver operating characteristic (ROC) curves for (**A**) pemphigus vulgaris, (**B**) mucous membrane pemphigoid, (**C**) bullous pemphigoid, and (**D**) linear IgA bullous dermatosis. Solid blue and red lines represent Claude Opus 4.7 and Gemini Pro 3, respectively; the dotted diagonal line represents chance-level discrimination (AUC = 0.50).

In the unadjusted paired DeLong comparisons, Claude demonstrated higher AUCs than Gemini for PV (*p* = 0.034), MMP (*p* = 0.010), and LABD (*p* = 0.035), whereas the difference for BP was not statistically significant (*p* = 0.156). After Holm adjustment for the four disease-specific AUC comparisons, only the difference for MMP remained statistically significant (adjusted *p* = 0.040); adjusted *p*-values were 0.102 for PV, 0.156 for BP, and 0.105 for LABD. Thus, although Claude showed numerically higher AUCs across all four disease categories, statistical evidence of a between-model difference after multiplicity adjustment was retained only for MMP (Figure 4). For PV, MMP, and BP, Claude achieved an AUC of exactly 1.000. Because an AUC of 1.000 results in zero estimated variance for Claude’s ROC curve, the corresponding DeLong comparisons have a degenerate variance component and their *p*-values should therefore be interpreted cautiously.

### 3.7. Calibration and Diagnostic Confidence

Calibration analyses demonstrated distinct probabilistic characteristics between the two models. Claude Opus 4.7 had a lower mean one-vs-rest Brier score than Gemini Pro 3 (0.0468 vs. 0.0558); however, the between-model difference was not statistically distinguishable from zero (difference, −0.0090; 95% CI, −0.0213 to 0.0020). The top-label Brier score was likewise slightly lower for Claude (0.1039 vs. 0.1092), with the corresponding between-model difference also including zero (−0.0053; 95% CI, −0.0267 to 0.0146). Thus, neither Brier analysis demonstrated a statistically significant between-model difference in probabilistic prediction error. In contrast, Gemini Pro 3 had substantially lower top-label expected calibration error (ECE) than Claude Opus 4.7 (0.0830 vs. 0.2514), with an observed between-model difference of 0.1684 (Claude − Gemini; 95% CI, 0.1115–0.2097), indicating lower top-label calibration error for Gemini. The maximum calibration error was also lower for Gemini (0.159 vs. 0.400) (Table 6).

**Figure 5:**
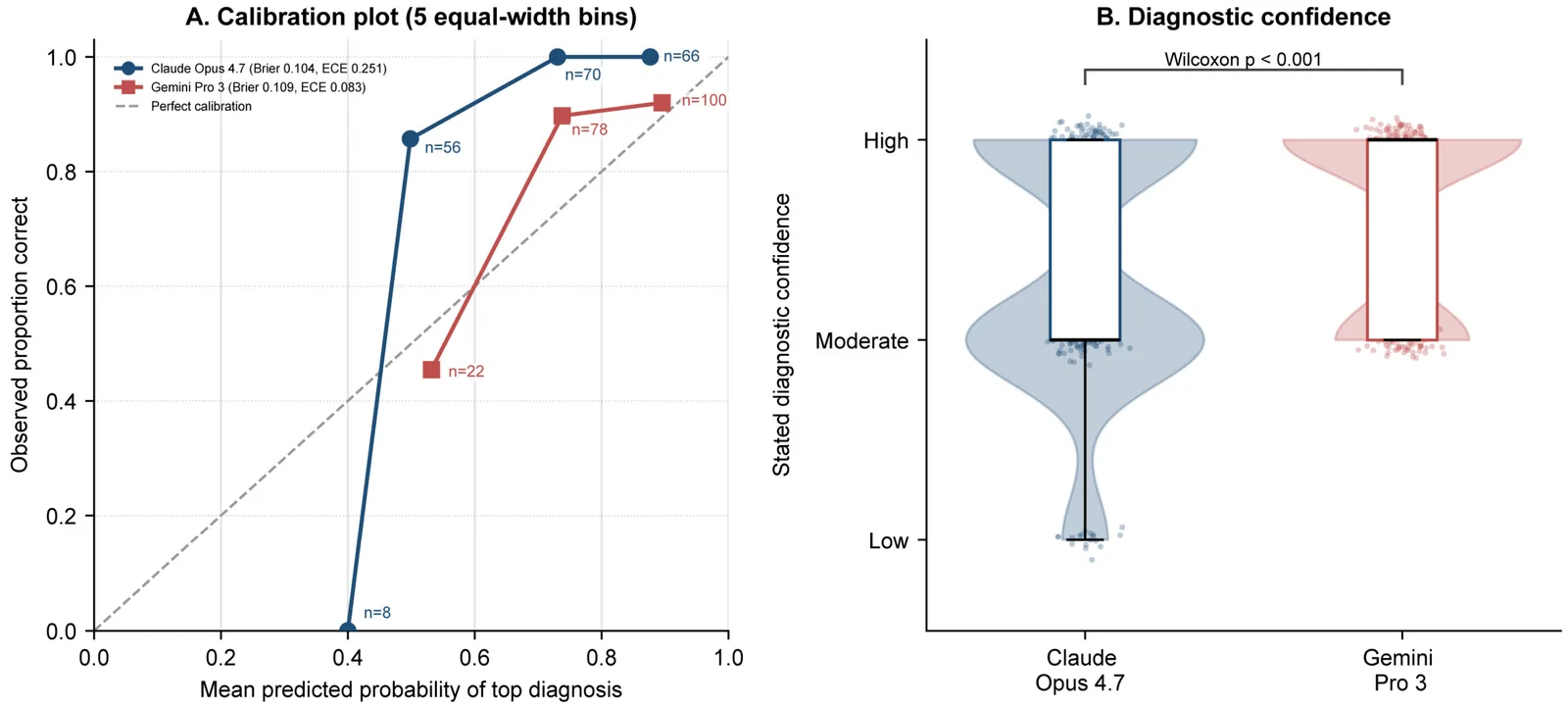
(**A**) Calibration plots of observed accuracy against mean predicted probability for the top-ranked diagnosis using five equal-width probability bins, with bin sizes annotated; the diagonal denotes perfect calibration. Points above the diagonal indicate under-confidence, whereas points below the diagonal indicate over-confidence. Blue and red represent Claude Opus 4.7 and Gemini Pro 3, respectively. (**B**) Violin and box plots of stated diagnostic confidence, with individual case observations shown as jittered dots; blue and red represent Claude Opus 4.7 and Gemini Pro 3, respectively.

Diagnostic confidence was reported as a three-level ordinal outcome according to the predefined confidence ranges specified in the prompt. Claude generated 18 low-confidence, 94 moderate-confidence, and 88 high-confidence responses, whereas Gemini generated no low-confidence responses, 72 moderate-confidence responses, and 128 high-confidence responses. The paired distributions of confidence categories differed significantly between the models (Wilcoxon signed-rank test, *p* < 0.001; r = 0.83). Confidence-stratified diagnostic accuracy for Claude was 22.2% among low-confidence responses, 97.9% among moderate-confidence responses, and 100.0% among high-confidence responses. For Gemini, diagnostic accuracy was 72.2% among moderate-confidence responses and 93.8% among high-confidence responses; no low-confidence Gemini responses were observed. The calibration plots and confidence distributions illustrating these findings are shown in Figure 5.

### 3.8. Clinical Reasoning and Management

Before consensus adjudication, the two assessors demonstrated substantial inter-rater agreement for key diagnostic-feature identification (Cohen’s κ = 0.912; 95% CI, 0.871–0.933), management appropriateness (Cohen’s κ = 0.844; 95% CI, 0.790–0.889), and clinical-reasoning quality (weighted Cohen’s κ = 0.88; 95% CI, 0.82–0.94). Following consensus adjudication, these consensus ratings were used for the comparative analyses reported above. Claude Opus 4.7 identified the key diagnostic features more accurately than Gemini Pro 3 (92.0% vs. 86.0%; McNemar *p* = 0.012), demonstrating superior extraction of clinically relevant diagnostic information. Management recommendations were appropriate in nearly all cases for both models (100.0% vs. 98.0%), with no statistically significant difference between them (*p* = 0.125). However, Claude generated higher-quality diagnostic reasoning than Gemini, with a greater proportion of responses rated as good (61.0% vs. 40.0%) and a higher overall reasoning score (mean ± SD: 2.61 ± 0.49 vs. 2.38 ± 0.53; median [IQR]: 3.0 [2.0–3.0] vs. 2.0 [2.0–3.0]; Wilcoxon signed-rank *p* < 0.001, *r* = 0.47), representing a moderate effect size. Notably, no Claude responses were rated as poor, whereas four Gemini responses received poor reasoning ratings (Table 7).

### 3.9. Error Pattern Analysis

Claude Opus 4.7 misclassified 16 of 200 cases (8.0%), whereas Gemini Pro 3 misclassified 28 cases (14.0%). The majority of errors were concentrated in linear IgA bullous dermatosis (LABD), indicating that this disease represented the greatest diagnostic challenge for both models. The predominant misclassification pattern was the erroneous classification of LABD as mucous membrane pemphigoid (MMP), accounting for 12 (75.0%) of Claude’s errors and 16 (57.1%) of Gemini’s errors (Table 8). This shared LABD → MMP error pattern represents the principal diagnostic challenge observed in the cohort. In contrast, bullous pemphigoid (BP) was classified correctly by both models in all cases, although Gemini generated two false-positive BP classifications among non-BP cases (both MMP), resulting in 98.7% BP specificity; Claude generated no false-positive BP classifications (Table 3).

**Figure 6:**
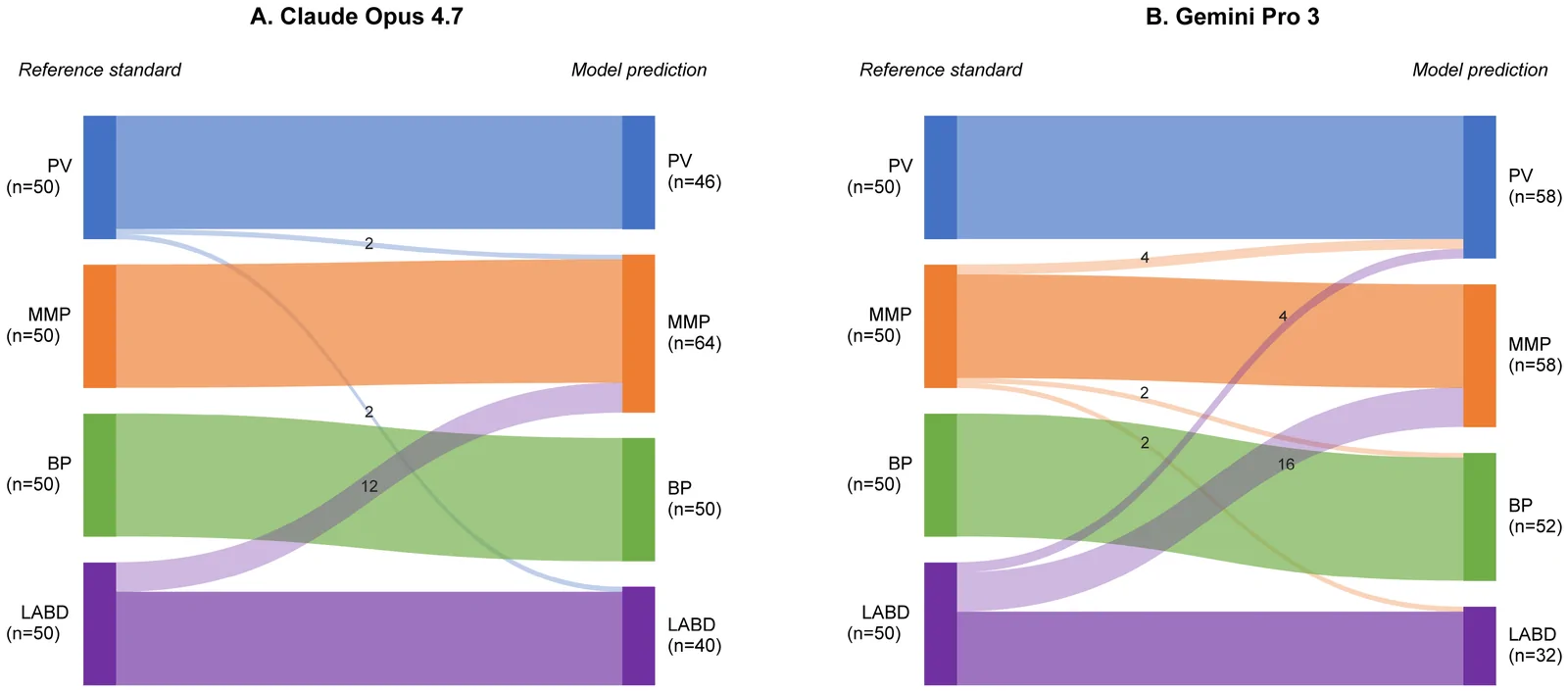
Alluvial (Sankey) diagram of diagnostic pathways for (**A**) Claude Opus 4.7 and (**B**) Gemini Pro 3. Ribbon width is proportional to case number; ribbons crossing between categories represent misclassifications, with the corresponding case counts indicated on the ribbons. The LABD → MMP stream represents the largest misclassification pathway in both panels.

Comparison of paired predictions showed that both models correctly classified 168 cases (84.0%), while Claude alone correctly diagnosed 16 cases (8.0%) that were misclassified by Gemini, compared with only 4 cases (2.0%) correctly identified by Gemini but missed by Claude. Both models misclassified the same 12 cases (6.0%), all representing LABD incorrectly classified as MMP, indicating a shared diagnostic challenge rather than a model-specific error pattern. The Sankey diagrams further demonstrate that the LABD → MMP pathway accounted for the majority of misclassifications in both models (Figure 6). Because histopathological and direct immunofluorescence findings were not provided to the AI models, these errors reflect limitations in diagnostic inference from the available clinical information and clinical photographs rather than failure to interpret immunopathological findings.

### 3.10. Multivariable and Sensitivity Analyses

To account for the paired study design, generalized estimating equations (GEE) were used to model the probability of a correct diagnosis while adjusting for disease category and within-case clustering. A binomial GEE with a logit link and exchangeable working correlation structure was fitted, with case specified as the clustering unit and Gemini Pro 3 and pemphigus vulgaris (PV) used as the reference categories. Bullous pemphigoid was excluded from the primary GEE model because both AI models achieved perfect classification, resulting in complete separation. A Firth penalized logistic regression including all four diseases was performed as a sensitivity analysis. Because this approach provides separation-robust estimates, unlike the GEE, it did not account for within-case clustering.

After adjustment for disease category, Claude Opus 4.7 demonstrated significantly higher odds of a correct diagnosis than Gemini Pro 3 (adjusted OR = 2.11, 95% CI: 1.24–3.59; *p* = 0.006). Disease category independently influenced diagnostic accuracy, with linear IgA bullous dermatosis (LABD) showing significantly lower odds of correct diagnosis than pemphigus vulgaris (adjusted OR = 0.077, 95% CI: 0.024–0.244; *p* < 0.001). The interaction between AI model and disease category was not significant (*p* = 0.953), indicating no statistical evidence that the association between AI model and diagnostic correctness differed across the included disease categories. Sensitivity analysis using Firth penalized logistic regression yielded comparable effect estimates, supporting the stability of the observed model effect under a separation-robust specification (Table 9).

## 4. Discussion

To our knowledge, this is the first multicentered paired accuracy study comparing two advanced multimodal large language models for the differential diagnosis of autoimmune bullous disease of the mouth (AIBD), namely Claude Opus 4.7 and Gemini Pro 3. The principal finding was that Claude Opus demonstrated significantly superior diagnostic accuracy, as well as superior clinical-reasoning performance compared with Gemini Pro 3. However, the observed differences should be interpreted within the controlled case mix and input configuration of the present study and should not be assumed to reflect intrinsic differences in model architecture or reasoning mechanisms [16].

Autoimmune blistering disorders necessitate interpretation of multiple types of heterogeneous information at the same time, which includes morphology of lesions, their distribution on the body, histopathology, results of direct immunofluorescence, and clinical history. In the present study, however, the AI models received clinical information and standardized clinical photographs only; histopathological and direct immunofluorescence (DIF) findings were used exclusively to establish the clinicopathological reference diagnosis. Therefore, the present results evaluate clinical-image-based diagnostic inference rather than multimodal interpretation of histopathological or DIF findings. Recent studies have shown that multimodal language models achieve better accuracy in diagnosis of skin conditions through incorporating both visual and text inputs into one diagnostic feature set compared with traditional image-based models. For instance, SkinGPT-4 proved the value of adding dermatologic images together with clinical and physician notes to improve the diagnostic accuracy [17].

The models also differed in their calibration and confidence characteristics. Claude demonstrated a lower one-vs-rest Brier score than Gemini, whereas Gemini demonstrated a lower expected calibration error (ECE). Thus, calibration performance was metric-dependent, and neither model can be characterized as uniformly superior in calibration. This distinction is clinically relevant because high diagnostic accuracy does not necessarily imply appropriately calibrated confidence. In particular, overconfident incorrect predictions may create greater clinical risk than appropriately uncertain predictions and should therefore be considered when evaluating LLMs for diagnostic support.

These results are consistent with growing research on how frontier multimodal LLMs exhibit high levels of competence in diagnosing disease through images. For instance, SkinGPT-4 in dermatology found that the integration of clinical metadata with images considerably improved the process of diagnostic reasoning and treatment suggestions [17]. Additionally, a recent study comparing the diagnostic performance of seven multimodal LLMs in dermatology using images found measurable differences in performance levels [18].

There is evidence from oral medicine that proves the worth of multimodal LLMs as clinical decision-making tools. The study conducted by Suárez et al. proved that ChatGPT-4o had good moderate diagnostic accuracy in diagnosing oral mucosal lesions, along with high accuracy in offering follow-up diagnostic procedures and treatment suggestions after making the right diagnosis. Hence, it can be said that multimodal LLMs can prove to be very helpful as adjunctive diagnostic instruments, but not as independent diagnostic instruments [19]. A systematic review published recently indicated that there was good potential shown by multimodal LLMs in diagnosing oral lesions; however, their diagnostic performance varied among different models [8].

Even though Claude performed better, both models demonstrated the same type of error, where linear IgA bullous dermatosis was incorrectly categorized as mucous membrane pemphigoid. This shared error is clinically relevant because LABD and MMP may exhibit overlapping oral clinical phenotypes. Importantly, DIF findings were not provided to either model; therefore, this error should not be interpreted as a failure to recognize or interpret a DIF pattern. Rather, it indicates the limitations of diagnostic inference from the clinical information and clinical photographic findings available to the models. Definitive immunopathological investigations remain important when clinically overlapping AIBDs cannot be reliably distinguished on clinical grounds alone.

This current study builds upon the growing body of research involving the application of multimodal LLMs to dermatologic and oral diseases by concentrating specifically on oral autoimmune blistering diseases, which have been identified as among the hardest-to-diagnose forms of oral mucosa disease due to the fact that diagnosis involves correlation between clinical features, histopathology, and direct immunofluorescence test results. The study specifically examines whether LLMs can infer the diagnosis from clinical information and clinical photographic findings when the definitive histopathological and immunopathological findings are withheld from the AI input. In contrast with other studies which have considered dermatologic conditions or a range of different oral lesions, this study directly contrasts two state-of-the-art multimodal LLMs by testing them under consistent clinical-image input and diagnostic accuracy criteria.

### 4.1. Diagnostic Performance in Context

In terms of diagnostic accuracy obtained by both models, they are much better than any previous studies on classification using classical machine learning techniques for AIBD. Singh et al. (2025) showed that accuracy rates of both classical machine learning and deep learning methods for autoimmune blistering skin disease classification range between 70% and 85%, while hybrid techniques show only slightly better results [20].

The AUC of 1.000 observed for PV, MMP, and BP with Claude indicates perfect separation within the present study cohort. However, these extreme AUC estimates should not be interpreted as evidence of perfect real-world discrimination. AUC estimates obtained from finite samples can reach 1.000 even when uncertainty remains around their generalizability, and their interpretation should therefore be accompanied by appropriate uncertainty assessment and external validation [21,22]. The balanced case distribution and relatively controlled case selection may also produce a case spectrum that differs from routine clinical practice, limiting transportability to more heterogeneous populations. External validation in independent cohorts with representative case spectra is therefore required before these discrimination estimates can be generalized to clinical practice [23,24]. Furthermore, the extreme calibration-slope estimates observed in association with quasi-complete separation should be interpreted cautiously because calibration slopes quantify the relationship between predicted and observed outcomes and can become unstable when predictions are highly separated.

The present study should be interpreted as an evaluation of investigational AI-based decision-support systems within a controlled, retrospective, closed-set four-class benchmark. It was not designed to establish autonomous medical diagnostic capability, prospective clinical effectiveness, patient benefit, or clinical safety.

### 4.2. Comparative Model Performance

Claude Opus 4.7 demonstrated higher comparative benchmark performance than Gemini Pro 3, with a 6.0-percentage-point difference in overall accuracy within the present experimental cohort. Claude achieved a 6.0-percentage-point higher overall diagnostic accuracy than Gemini, with 16 discordant pairs favoring Claude and 4 favoring Gemini. This paired difference indicates better performance by Claude within the present cohort; however, its clinical magnitude should be interpreted in the context of the study design, case spectrum, and uncertainty around the effect estimate.

The difference in performance between the two models was more significant in difficult-to-diagnose cases, namely LABD and MMP. Claude had significantly higher sensitivity for LABD (76.0% vs. 60.0%) and perfect sensitivity for MMP (100.0% vs. 84.0%). This can be explained by the diagnostic difficulties of the aforementioned diseases due to their overlapping symptoms and need to integrate immunopathological findings for proper differential diagnosis [4]. Because immunopathological findings were not provided to the models, the observed differences reflect their ability to infer diagnoses from the available clinical information and photographic phenotype rather than their ability to integrate DIF or histopathological findings. Despite the proprietary nature of these models that prevents any definitive explanations regarding mechanisms of their functioning, such variations can be attributed to differing levels of multimodal reasoning, response calibration, and clinical-information and visual-feature integration.

### 4.3. Diagnostic Errors and Clinical Implications

The most frequent error pattern involving misdiagnosis of LABD as MMP constituted 75.0% of Claude’s errors and 57.1% of Gemini’s errors. This error pattern is consistent with the recognized diagnostic difficulty of distinguishing these diseases because of overlapping clinical manifestations [25,26]. Importantly, histopathological and DIF findings were not provided to either AI model and were used exclusively to establish the clinicopathological reference diagnosis. Therefore, the shared LABD → MMP error reflects a limitation of diagnostic inference from clinical information and clinical photographic findings rather than a failure to interpret immunopathological evidence. Distinguishing these entities may require direct immunofluorescence assessment of immunoglobulin and complement deposition patterns and, when appropriate, disease-specific serological testing [5]. These findings reinforce the importance of clinicopathological and immunopathological confirmation when clinically overlapping AIBDs are suspected, rather than relying on LLM-based inference alone.

Management recommendations met the predefined appropriateness criteria in 100.0% of Claude responses and 98.0% of Gemini responses; however, agreement with predefined expert criteria in this retrospective experimental setting does not establish the safety or effectiveness of AI-generated management recommendations. The assessment did not evaluate contraindications, drug interactions, longitudinal outcomes, adverse events, or the consequences of acting on an incorrect recommendation. Management suggestions may also vary with diagnostic certainty, disease severity, comorbidities, concomitant medications, and other patient-specific factors. Accordingly, AI-generated management suggestions should be treated as adjunctive information requiring independent clinician verification and contextual assessment before influencing patient care. Clinical implementation would require a supervised workflow, appropriate clinicopathological and immunopathological verification where indicated, and safeguards against erroneous, hallucinated, or overconfident outputs and automation bias. Prospective human-in-the-loop evaluation and formal safety assessment would be required before routine clinical use.

### 4.4. Calibration and Clinical Confidence

The calibration results were not uniformly favorable for either model. Claude demonstrated a lower one-vs-rest Brier score (0.0468 vs. 0.0558), whereas Gemini demonstrated a lower ECE (0.083 vs. 0.251). These findings illustrate that calibration conclusions depend on the metric used and that diagnostic accuracy and confidence calibration are distinct properties. In addition, the extreme AUC values and associated quasi-complete separation observed for some Claude disease categories produced unstable calibration-slope estimates. These estimates should therefore not be interpreted as clinically meaningful measures of calibration [13].

Given the observed association between Claude confidence and diagnostic correctness, confidence outputs may provide exploratory signals for identifying cases warranting additional scrutiny. However, high model confidence should not be regarded as clinical reassurance or used independently to guide clinical decisions. Incorrect high-confidence predictions remain possible, and confidence should therefore be interpreted alongside diagnostic evidence and, when indicated, definitive investigations [12].

### 4.5. Clinical Reasoning and Integration

Claude also exhibited significantly better quality of clinical reasoning (61.0% of answers were good versus 40.0% for Gemini; Wilcoxon signed-rank *p* < 0.001, r = 0.47), and the ability to recognize key diagnostic criteria was better (92.0% versus 86.0%; *p* = 0.012). These findings were based on assessments performed independently by two board-certified oral medicine specialists, with substantial pre-consensus inter-rater agreement for key diagnostic-feature identification, management appropriateness, and clinical-reasoning quality. Coherent explanations of diagnostic reasoning are crucial in ensuring clinical acceptance, since clinicians need accurate diagnoses along with logical and justified explanation that can be incorporated in their clinical reasoning [8,27].

The results are consistent with the recent literature on the diagnostic potential of multimodal LLMs in the field of oral medicine. In 2026, Robaian et al. showed that multimodal LLMs can provide diagnostic subtyping and risk assessment for oral lichen planus with the accuracy that is comparable with the one of expert clinicians [28]. These findings should be interpreted as evaluation of the quality and structure of model-generated reasoning outputs under the study protocol, rather than evidence that the models reproduce expert clinical reasoning or can independently support clinical decisions.

### 4.6. Comparison with Existing AI Approaches

Our results add to the rapidly expanding pool of knowledge in the area of application of AI for autoimmune diseases. Mahajan et al. (2025) gave a complete review of AI applications for autoimmune diseases, emphasizing the capabilities of AI for improving diagnosis and predicting response to therapy as well as identifying new subgroups of the disease [29]. Likewise, Manuelyan et al. (2024) focused on the AI applications in autoimmune bullous dermatoses and emphasized difficulties associated with the lack of datasets and the need for a multimodal approach [30].

Li et al. (2024) showed the feasibility of human-multimodal deep learning collaboration for accurate diagnosis of different lupus erythematosus variants and other similar skin conditions with diagnostic accuracy comparable to that of dermatology specialists [31]. The current study extends this literature to oral AIBDs and provides evidence that multimodal LLMs can achieve high diagnostic performance under standardized evaluation conditions; however, the findings do not establish that standalone LLM use is equivalent or superior to specialist clinical assessment.

These findings further support growing evidence that multimodal integration is central to improving AI performance in complex autoimmune diseases [32].

### 4.7. Study Limitations

Several limitations should be acknowledged. First, the balanced dataset (25% per disease) does not reflect real-world AIBD prevalence; therefore, predictive values should be interpreted as a benchmark rather than population-specific estimates. The controlled case distribution may also contribute to extreme discrimination estimates, requiring external validation in prevalence-representative cohorts.

Second, the retrospective use of archival cases may introduce selection bias because cases with complete documentation and adequate-quality photographs may not represent the full clinical spectrum. A single clinical photograph may also incompletely capture lesion distribution and morphology, potentially affecting diagnostic performance.

Third, histopathological and DIF findings were not provided to the AI models but were used to establish the reference diagnosis; thus, the study assessed clinical-image-based inference rather than immunopathological image interpretation. This may be particularly relevant to overlapping entities such as LABD and MMP.

Fourth, real-world performance may differ with variations in case complexity, image acquisition, available information, and model interaction. Only two models were evaluated at a single time point, and provider-side model or interface updates may affect reproducibility; moreover, web-based testing did not permit investigator control of certain generation parameters or immutable backend snapshots.

Fifth, clinical reasoning, key-feature identification, and management assessment involved expert judgment, although pre-consensus inter-rater reliability was formally assessed. No direct clinician–AI comparison was performed; therefore, equivalence or superiority to expert judgment cannot be inferred.

Sixth, the models were evaluated within a controlled, closed-set four-class benchmark and were not tested as autonomous clinical decision-makers. The study therefore provides no evidence regarding prospective clinical effectiveness, patient benefit, treatment safety, or the consequences of acting on incorrect or hallucinated outputs. Potential clinical implementation would require independent clinician verification of model-generated diagnostic and management outputs, safeguards against overconfidence and automation bias, and prospective human-in-the-loop safety evaluation.

Finally, the 200 paired cases provided a reasonably precise estimate of the 6.0-percentage-point primary accuracy difference, but the post-hoc sensitivity analysis indicated approximately 73% power under the observed discordance structure. The findings should therefore be interpreted primarily as comparative effect estimates rather than definitive evidence that smaller differences would be detected.

### 4.8. Future Directions

These aspects can be subject to future research efforts. Prospective validation studies conducted in the context of real clinical practice are crucial for verification of results and identification of practical issues related to implementation of these technologies. Future studies should use independent, prevalence-representative cohorts with broader clinical spectra, including atypical and diagnostically challenging cases, and should prospectively evaluate calibration and clinically relevant error patterns. Studies devoted to the analysis of interaction models between humans and AI, where clinical specialists receive assistance from machine predictions, may contribute to identification of best practices for model implementation [10]. Analysis of the model performance among various patient groups, levels of severity of diseases and in different settings is necessary for improving generalizability of the results. In particular, future work should compare AI-only, clinician-only, and clinician–AI collaborative diagnostic performance and should evaluate whether access to histopathological and DIF information improves discrimination of clinically overlapping AIBDs such as LABD and MMP.

Prospective studies should also evaluate whether calibrated uncertainty, rather than diagnostic accuracy alone, can safely guide referral for additional investigations and specialist review.

## 5. Conclusions

This study demonstrates high diagnostic performance of multimodal LLMs for oral AIBDs under standardized paired evaluation, with Claude Opus 4.7 outperforming Gemini Pro 3 in overall accuracy and clinical-reasoning performance. Calibration was metric-dependent, and extreme AUCs should be interpreted cautiously given the balanced retrospective cohort and separation-related instability. Neither model received histopathological or DIF findings, which were used exclusively to establish the reference diagnosis. The shared LABD → MMP misclassification highlights the limitations of clinical-image-based inference for overlapping AIBDs. LLMs should therefore be used as adjunctive decision-support tools, with clinician oversight and appropriate diagnostic verification. These findings represent controlled benchmark performance rather than evidence of autonomous diagnostic capability, clinical effectiveness, or safety. The models should therefore be regarded as investigational adjunctive decision-support tools requiring clinician oversight and appropriate diagnostic verification. Prospective external and human-in-the-loop validation is required before clinical implementation.

## Figures and Tables

**Table 1:** Composition of the study cohort and completeness of model outputs (*n* = 200 cases).

Characteristic	Cases, *n* (%)	Claude Opus 4.7 Evaluable, *n* (%)	Gemini Pro 3 Evaluable, *n* (%)	Reference Standard
Pemphigus vulgaris (PV)	50 (25.0)	50 (100)	50 (100)	Histopathology + DIF
Mucous membrane pemphigoid (MMP)	50 (25.0)	50 (100)	50 (100)	Histopathology + DIF
Bullous pemphigoid (BP)	50 (25.0)	50 (100)	50 (100)	Histopathology + DIF
Linear IgA bullous dermatosis (LABD)	50 (25.0)	50 (100)	50 (100)	Histopathology + DIF
Interpretable top diagnosis	200 (100)	200 (100)	200 (100)	—
Complete probability vector	200 (100)	200 (100)	200 (100)	—
Indeterminate/non-response	0 (0)	0 (0)	0 (0)	—

DIF, direct immunofluorescence. Percentages are column percentages of the 200-case analysis set. The balanced design fixes disease prevalence at 25% per category; predictive values below should be interpreted accordingly.

**Table 2:** Overall diagnostic performance of Gemini Pro 3 and Claude Opus 4.7 against the reference standard (*n* = 200 paired cases).

Metric	Claude Opus 4.7	Gemini Pro 3	Between-Model Comparison
Correct primary diagnoses, *n*/N	184/200	172/200	—
Overall accuracy, % (Wilson 95% CI)	92.0 (87.4–95.0)	86.0 (80.5–90.1)	Δ = 6.0% (95% CI: 1.7–10.3)
Overall accuracy, bootstrap 95% CI	87.5–95.5	80.8–90.7	Bootstrap Δ: consistent with analytical estimate
Balanced accuracy, %	92.0	86.0	—
Cohen’s κ vs. reference (95% CI)	0.893 (0.843–0.943)	0.813 (0.750–0.877)	—
Discordant pairs (Claude correct/Gemini incorrect)	16	—	McNemar *p* = 0.012
Discordant pairs (Gemini correct/Claude incorrect)	—	4	Paired OR = 4.00 (95% CI: 1.29–16.44)
Risk difference, % (95% CI)	—	—	6.0 (1.7–10.3)
Number needed to diagnose (NND)	—	—	16.7
Cohen’s h	—	—	0.19 (small)
Inter-model agreement, %	—	—	90.0
Inter-model Cohen’s κ (95% CI)	—	—	0.866 (0.810–0.921)

CI, confidence interval; κ, Cohen’s kappa; OR, odds ratio; NND, number needed to diagnose. Wilson score confidence intervals are reported for proportions and were corroborated by percentile bootstrap confidence intervals based on 10,000 paired case-level resamples. McNemar’s exact test was used for paired comparisons. The paired odds ratio was calculated from discordant pairs using exact confidence intervals. Cohen’s h quantifies the standardized difference in proportions (0.2 = small, 0.5 = medium, 0.8 = large). The primary analysis used the intentionally balanced 25% disease distribution. Prevalence-weighted estimates were evaluated separately as a sensitivity analysis and are reported in Supplementary Table S1.

**Table 3:** Disease-specific diagnostic performance of both models (one-vs-rest; 50 positive cases and 150 negative cases per disease).

Disease	Model	Sensitivity% (Wilson 95% CI)	Sensitivity% (Bootstrap 95% CI)	Specificity% (Wilson 95% CI)	F1-Score	Balanced Accuracy%
PV	Claude Opus 4.7	92.0 (81.2–96.8)	83.7–98.2	100.0 (97.5–100.0)	0.958	96.0
	Gemini Pro 3	100.0 (92.9–100.0)	100.0–100.0	94.7 (89.8–97.3)	0.926	97.3
MMP	Claude Opus 4.7	100.0 (92.9–100.0)	100.0–100.0	90.7 (84.9–94.4)	0.877	95.3
	Gemini Pro 3	84.0 (71.5–91.7)	73.8–93.3	89.3 (83.4–93.3)	0.778	86.7
BP	Claude Opus 4.7	100.0 (92.9–100.0)	100.0–100.0	100.0 (97.5–100.0)	1.000	100.0
	Gemini Pro 3	100.0 (92.9–100.0)	100.0–100.0	98.7 (95.3–99.6)	0.980	99.3
LABD	Claude Opus 4.7	76.0 (62.6–85.7)	63.6–87.2	98.7 (95.3–99.6)	0.844	87.3
	Gemini Pro 3	60.0 (46.2–72.4)	46.5–72.9	98.7 (95.3–99.6)	0.732	79.3

Bootstrap intervals are percentile intervals from 10,000 case-level resamples, which preserve the pairing of the two models within each case. Wilson and bootstrap intervals were broadly consistent across the reported performance measures. F1 is the harmonic mean of precision and recall. Where an estimate is 100%, the Wilson interval is one-sided by construction.

**Table 4:** Multiclass classification performance metrics (four-class task, *n* = 200 cases per model).

Metric	Claude Opus 4.7	Gemini Pro 3	Difference
Accuracy (micro-averaged F1), %	92.0	86.0	6.0
Macro-averaged F1	0.920	0.854	0.066
Weighted F1	0.920	0.854	0.066
Macro-averaged precision	0.933	0.871	0.062
Macro-averaged recall	0.920	0.860	0.060
Weighted precision	0.933	0.871	0.062
Weighted recall	0.920	0.860	0.060
Balanced accuracy, %	92.0	86.0	6.0

In this balanced four-class design, micro-averaged F1 equals overall accuracy, and weighted F1, precision, and recall are numerically identical to their corresponding macro-averaged metrics because each diagnostic category contributes equally to the dataset.

**Table 5:** One-versus-rest receiver operating characteristic analysis of Claude Opus 4.7 and Gemini Pro 3.

Disease	Claude Opus 4.7 AUC (95% CI)	Gemini Pro 3 AUC (95% CI)	ΔAUC	*p* Value (DeLong)	Holm-Adjusted *p*
PV	1.000 (1.000–1.000)	0.992 (0.985–0.999)	0.008	0.034	0.102
MMP	1.000 (1.000–1.000)	0.929 (0.875–0.983)	0.071	0.010	0.040
BP	1.000 (1.000–1.000)	0.987 (0.968–1.000)	0.013	0.156	0.156
LABD	0.993 (0.985–1.000)	0.952 (0.914–0.990)	0.041	0.035	0.105
Macro-average AUC	0.998	0.965	0.033	—	

AUC, area under the receiver operating characteristic curve; ΔAUC, Claude Opus 4.7 AUC minus Gemini Pro 3 AUC; CI, confidence interval. AUCs were calculated using one-versus-rest predicted probabilities for each disease category, with the remaining disease categories treated as the negative class. Confidence intervals were calculated using DeLong’s method with logit-scale transformation. *p*-values are from paired DeLong comparisons and Holm-adjusted across the four disease-specific AUC comparisons. Macro-average AUC represents the unweighted mean of the four one-versus-rest AUCs. For Claude, AUC = 1.000 for PV, MMP, and BP; consequently, the estimated DeLong variance for Claude is zero for these comparisons, and the corresponding *p*-values should be interpreted cautiously.

**Table 6:** Calibration metrics and confidence-stratified diagnostic accuracy of Claude Opus 4.7 and Gemini Pro 3.

Metric	Claude Opus 4.7	Gemini Pro 3	Between-Model Comparison
**[Object]**			
Brier score, one-vs-rest mean	0.0468 (95% CI, 0.0400–0.0539)	0.0558 (95% CI, 0.0428–0.0700)	−0.0090 (95% CI, −0.0213 to 0.0020)
Brier score, top prediction	0.1039 (95% CI, 0.0913–0.1168)	0.1092 (95% CI, 0.0863–0.1345)	−0.0053 (95% CI, −0.0267 to 0.0146)
Expected calibration error (5 equal-width bins)	0.2514 (95% CI, 0.2228–0.2791)	0.0830 (95% CI, 0.0523–0.1267)	0.1684 (95% CI, 0.1115–0.2097)
Maximum calibration error	0.400	0.159	
**Diagnostic confidence *n* (%)**			
Low	18 (9.0%)	0 (0%)	Wilcoxon *p* ≤ 0.001
Moderate	94 (47.0%)	72 (36.0%)	Effect size r = 0.83
High	88 (44.0%)	128 (64.0%)	
**Accuracy within confidence level (%)**			
Low confidence	22.2 (9.0–45.2)	*n* = 0	—
Moderate confidence	97.9 (92.6–99.4)	72.2 (61.0–81.2)	—
High confidence	100.0 (95.8–100.0)	93.8 (88.2–96.8)	—

The 95% confidence intervals for Brier scores and ECE were obtained using 10,000 paired case-level bootstrap resamples, with identical resampled case indices applied to both models to preserve pairing. ECE was calculated using five equal-width probability bins spanning [0, 1]. Brier score and ECE assess different properties of probabilistic predictions and should not be interpreted interchangeably. Claude’s calibration slope and intercept were not interpreted because of quasi-complete separation. Effect size r = Z/√*n* for the Wilcoxon signed-rank test.

**Table 7:** Clinical reasoning, key diagnostic feature identification, and management recommendations of Claude Opus 4.7 and Gemini Pro 3 (*n* = 200 paired cases).

Outcome	Claude Opus 4.7	Gemini Pro 3	Between-Model Comparison
**Key diagnostic features correctly identified**			
Discordant pairs (Claude+/Gemini−; Claude−/Gemini+)	184/200 (92.0 (87.4–95.0))	172/200 (86.0 (80.5–90.1))	McNemar *p* = 0.012
Discordant pairs (b/c)	16	4	—
**Appropriate management recommendations**			
*n*/N (%, 95% CI)	200/200 (100.0 (98.1–100.0))	196/200 (98.0 (95.0–99.2))	McNemar *p* = 0.125
Discordant pairs (Claude+/Gemini−; Claude−/Gemini+)	4	0	—
**Reasoning quality score**			
Mean ± SD	2.61 ± 0.49	2.38 ± 0.53	Wilcoxon *p* < 0.001
Median [IQR]	3.0 [2.0–3.0]	2.0 [2.0–3.0]	Effect size r = 0.47
Poor/Acceptable/Good, *n*	0/78/122	4/116/80	—
Responses rated good, % (95% CI)	61.0 (54.1–67.5)	40.0 (33.5–46.9)	—
**Pre-consensus inter-rater reliability**			
Key diagnostic-feature identification		Cohen’s κ = 0.912 (95% CI, 0.871–0.933)	
Management appropriateness		Cohen’s κ = 0.844 (95% CI, 0.790–0.889)	
Clinical reasoning quality		Weighted Cohen’s κ = 0.88 (95% CI, 0.82–0.94)	

Discordant pair b denotes cases where Claude was correct and Gemini was not; c denotes the converse. McNemar tests use the exact binomial form given fewer than 25 discordant pairs. The management comparison is based on only four discordant pairs and is therefore underpowered; the non-significant result should not be read as evidence of equivalence. Inter-rater reliability was assessed before consensus adjudication using Cohen’s κ for binary outcomes and weighted Cohen’s κ for the three-level reasoning-quality score.

**Table 8:** Diagnostic error patterns and concordance analysis of Claude Opus 4.7 and Gemini Pro 3.

**A. Misclassification Pathways**			
**Reference-Standard Diagnosis**	**Misclassified as**	**Claude Opus 4.7, *n* (%)**	**Gemini Pro 3, *n* (%)**
LABD	MMP	12 (24.0)	16 (32.0)
LABD	PV	0	4 (8.0)
PV	MMP	2 (4.0)	0
PV	LABD	2 (4.0)	0
MMP	PV	0	4 (8.0)
MMP	BP	0	2 (4.0)
MMP	LABD	0	2 (4.0)
Total misclassified cases	—	16 (8.0)	28 (14.0)
**B. Concordance of diagnostic outcomes**			
**Outcome**	***n***	**% of 200 cases**	**Interpretation**
Both models correct	168	84.0	Concordant success
Claude correct, Gemini incorrect	16	8.0	Favours Claude
Gemini correct, Claude incorrect	4	2.0	Favours Gemini
Both models incorrect	12	6.0	Shared failure mode

Percentages for misclassification pathways are calculated using the 50 reference-standard cases within each disease category. Only non-zero misclassification pathways are presented. The 12 concordant errors involved LABD misclassified as MMP by both models, indicating a shared diagnostic challenge.

**Table 9:** Population-averaged GEE and Firth penalized logistic regression models of correct diagnosis by AI model and disease category.

Term	Primary Analysis (GEE) Adjusted OR (95% CI)	*p* Value	Sensitivity Analysis (Firth) Adjusted OR (95% CI)	*p* Value
Claude Opus 4.7 vs. Gemini Pro 3	2.11 (1.24–3.59)	0.006	2.07 (1.04–4.12)	0.040
MMP vs. PV	0.41 (0.12–1.38)	0.150	0.51 (0.16–1.65)	0.258
LABD vs. PV	0.08 (0.02–0.24)	<0.001	0.10 (0.03–0.27)	<0.001
BP vs. PV			9.41 (0.50–176.92)	0.134

BP was excluded from the primary GEE model because both AI models correctly classified all BP cases, resulting in complete separation. The Firth penalized logistic regression included all four disease categories and was performed as a separation-robust sensitivity analysis. The GEE used an exchangeable working correlation structure with case as the clustering unit; the Firth model did not account for within-case clustering. These adjusted analyses were secondary and were intended to estimate the association between model type and diagnostic correctness after adjustment for disease category, rather than replace the prespecified paired McNemar analysis.

## Data Availability

Research data supporting this publication is available from the corresponding author upon request.

## Supplementary Materials

The following supporting information can be downloaded at: https://www.mdpi.com/article/10.3390/diagnostics16172761/s1, Figure S1: Representative Clinical Cases Across the Four Autoimmune Blistering Diseases; Part S1: Standardized Evaluation Prompt; Table S1: Sensitivity analysis of overall diagnostic accuracy under alternative disease distributions.

## Abbreviations

The following abbreviations are used in this manuscript:

**Abbreviation**	**Definition**
AI	Artificial Intelligence
AIBD	Autoimmune Blistering Disease
AUC	Area Under the Receiver Operating Characteristic Curve
BP	Bullous Pemphigoid
CI	Confidence Interval
DIF	Direct Immunofluorescence
ECE	Expected Calibration Error
ELISA	Enzyme-Linked Immunosorbent Assay
F1-score	Harmonic Mean of Precision and Recall
GEE	Generalized Estimating Equation
IIF	Indirect Immunofluorescence
IQR	Interquartile Range
κ	Cohen’s Kappa Coefficient
LABD	Linear IgA Bullous Dermatosis
LLM	Large Language Model
MLLM	Multimodal Large Language Model
MMP	Mucous Membrane Pemphigoid
NND	Number Needed to Diagnose
OR	Odds Ratio
PV	Pemphigus Vulgaris
ROC	Receiver Operating Characteristic
SD	Standard Deviation
STARD	Standards for Reporting Diagnostic Accuracy Studies
STARD-AI	Standards for Reporting Diagnostic Accuracy Studies–Artificial Intelligence Extension
Wilson CI	Wilson Confidence Interval
