# Supplementary material for: Diagnostic Accuracy of Multimodal Large Language Models for Four-Class Benchmark of Oral Autoimmune Blistering Diseases: A Multicenter Paired Study
Source: Diagnostics (Basel). 2026 Aug 28;16(17):2761. doi: 10.3390/diagnostics16172761 (PMC13564754; doi:10.3390/diagnostics16172761)
Supplement: Supplementary file 1 [file diagnostics-16-02761-s001.zip › diagnostics-4492516-supplementary.pdf]

Supplementary Figure S1. Representative Clinical Cases Across the Four Autoimmune Blistering Diseases

Case	Patient demographics	Relevant medical history	Clinical presentation	Clinical photographs	Reference diagnosis
Case 1	62-year-old male	No significant systemic disease. No recent medication changes. No known autoimmune disease.	**Duration:** 2 months. **Symptoms:** painful oral erosions and burning, aggravated by eating. **Oral involvement:** Extensive erosions involving the buccal and labial mucosa, gingiva, and lips. **Morphology:** irregular, shallow erosions with surrounding erythema, epithelial sloughing, and areas of hemorrhagic crusting. **Extraoral:** erosive crusted lesion on the skin of the forearm.	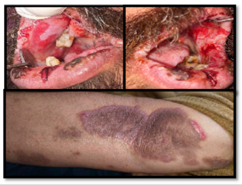	**Pemphigus vulgaris (PV)**
Case 2	53-year-old female	Hypertension controlled with medication; no previously diagnosed autoimmune disease.	**Duration:** 5 months. **Symptoms:** recurrent painful oral ulceration and soreness. **Oral involvement:** bilateral buccal mucosa, vestibular mucosa, hard palate, lateral tongue, and floor of mouth. **Morphology:** erythematous erosions and ulcerations with localized mucosal atrophy and epithelial disruption. **Extraoral:** no significant cutaneous lesions reported.	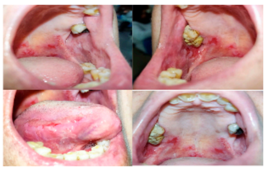	**Mucous membrane pemphigoid (MMP)**
Case 3	32-year-old male	Type 2 diabetes mellitus and hypertension; no previous autoimmune blistering disease.	**Duration:** 6 weeks. **Symptoms:** painful lesions with perioral irritation and swelling. **Clinical involvement:** erythematous, crusted erosive lesions involving the perioral skin and vermilion border, extending from the oral commissure onto adjacent perioral skin. **Morphology:** erythematous erosions with hemorrhagic crusting and lip swelling.	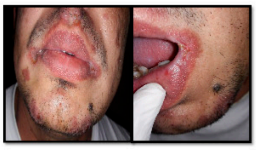	**Bullous pemphigoid (BP)**
Case 4	48-year-old female	No significant systemic disease; no known autoimmune disorder.	**Duration:** 3 weeks. **Symptoms:** painful oral erosions and mucosal sensitivity. **Oral involvement:** maxillary labial mucosa and gingiva. **Morphology:** erythematous erosive lesion with epithelial sloughing and a translucent partially detached epithelial layer. **Extraoral:** no significant cutaneous involvement reported.	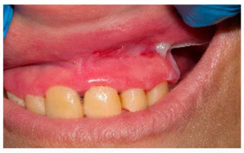	**Linear IgA bullous dermatosis (LABD)**

Supplementary Part S1. Standardized Evaluation Prompt

The following standardized prompt was used for each case:

You are an expert in oral medicine and dermatology. Analyze the following clinical case information and clinical photograph. Based on the available clinical features and history, determine the most likely diagnosis among the following four autoimmune blistering diseases:

1. Pemphigus vulgaris (PV)

2. Mucous membrane pemphigoid (MMP)

3. Bullous pemphigoid (BP)

4. Linear IgA bullous dermatosis (LABD)

Please provide:

(1) The most likely diagnosis.

(2) A probability (%) for each of the four diagnostic categories. The four probabilities must sum to 100%.

(3) Diagnostic confidence as one of the following predefined categories:

- **Low:** 0–49%
- **Moderate:** 50–79%
- **High:** 80–100%

(4) The key clinical features supporting the diagnostic assessment.

(5) An appropriate initial management plan.

(6) Clearly state relevant uncertainty or diagnostic limitations when appropriate.

Supplementary Table S1. Sensitivity analysis of overall diagnostic accuracy under alternative disease distributions

Weighting scenario	Claude, %	Gemini, %	Δ, percentage points	95% CI for Δ
Primary analysis—balanced study design (25% each)	92.0	86.0	+6.0	1.7 to 10.3
Illustrative clinical distribution 1 (BP 45%, LABD 5%, PV 25%, MMP 25%)	96.8	94.0	+2.8	−0.4 to 6.0
Illustrative clinical distribution 2 (BP 35%, LABD 5%, PV 30%, MMP 30%)	96.4	93.2	+3.2	−0.6 to 7.0

Weighted accuracies were calculated by direct standardization of the observed disease-specific accuracies to the specified disease distributions. The 95% CIs for the between-model differences were estimated from the within-disease discordant-pair variance. The primary balanced analysis reproduces the prespecified paired analysis reported in the main manuscript. The alternative disease distributions are illustrative and were not derived from a single epidemiological registry; therefore, these estimates represent sensitivity analyses of generalizability rather than population-level clinical accuracy.
